# Supplementary material for: Modeling glioblastoma heterogeneity as a dynamic network of cell states
Source: Mol Syst Biol. 2021 Sep 16;17(9):e10105. doi: 10.15252/msb.202010105 (PMC8444284; doi:10.15252/msb.202010105)
Supplement: Supplementary file 6 — Source Data for Figure 5 [file MSB-17-e10105-s004.zip › Figure5A_sourcedata/GSEA_3071/nosigdb_stateIII.GseaPreranked.1622795155562/RICHARDS_NOWAKOWSKI_SCIENCE_2017_GLYC_UPREG.html]

Details for gene set RICHARDS\_NOWAKOWSKI\_SCIENCE\_2017\_GLYC\_UPREG[GSEA]

|  || Dataset | state33071 |
| Phenotype | NoPhenotypeAvailable |
| Upregulated in class | na\_pos |
| GeneSet | RICHARDS\_NOWAKOWSKI\_SCIENCE\_2017\_GLYC\_UPREG |
| Enrichment Score (ES) | 0.10031127 |
| Normalized Enrichment Score (NES) | 1.385391 |
| Nominal p-value | 0.0 |
| FDR q-value | 0.09965739 |
| FWER p-Value | 0.719 |
Table: GSEA Results Summary

  

Fig 1: Enrichment plot: RICHARDS\_NOWAKOWSKI\_SCIENCE\_2017\_GLYC\_UPREG      
 Profile of the Running ES Score & Positions of GeneSet Members on the Rank Ordered List

  

| PROBE | GENE SYMBOL | GENE\_TITLE | RANK IN GENE LIST | RANK METRIC SCORE | RUNNING ES | CORE ENRICHMENT || 1 | EIF2S2 |  |  | 30 | 0.499 | -0.0026 | Yes |
| 2 | NACA |  |  | 53 | 0.459 | -0.0034 | Yes |
| 3 | NSA2 |  |  | 56 | 0.456 | 0.0011 | Yes |
| 4 | ZFAS1 |  |  | 65 | 0.447 | 0.0039 | Yes |
| 5 | RPS7 |  |  | 75 | 0.436 | 0.0064 | Yes |
| 6 | PSMA7 |  |  | 78 | 0.435 | 0.0107 | Yes |
| 7 | C6orf48 |  |  | 81 | 0.430 | 0.0149 | Yes |
| 8 | EIF1 |  |  | 86 | 0.426 | 0.0186 | Yes |
| 9 | NPM1 |  |  | 88 | 0.421 | 0.0230 | Yes |
| 10 | VDAC1 |  |  | 98 | 0.413 | 0.0251 | Yes |
| 11 | FAU |  |  | 113 | 0.400 | 0.0258 | Yes |
| 12 | DRAP1 |  |  | 116 | 0.399 | 0.0297 | Yes |
| 13 | SEC61G |  |  | 118 | 0.397 | 0.0339 | Yes |
| 14 | NDUFA4 |  |  | 140 | 0.382 | 0.0324 | Yes |
| 15 | RPL36AL |  |  | 144 | 0.381 | 0.0359 | Yes |
| 16 | MAP1LC3B |  |  | 154 | 0.370 | 0.0375 | Yes |
| 17 | SSBP1 |  |  | 156 | 0.370 | 0.0414 | Yes |
| 18 | RPL18 |  |  | 178 | 0.355 | 0.0397 | Yes |
| 19 | GAS5 |  |  | 179 | 0.355 | 0.0436 | Yes |
| 20 | RPS27L |  |  | 188 | 0.348 | 0.0453 | Yes |
| 21 | NHP2 |  |  | 232 | 0.322 | 0.0373 | Yes |
| 22 | BUD31 |  |  | 237 | 0.316 | 0.0397 | Yes |
| 23 | NDUFA2 |  |  | 239 | 0.315 | 0.0429 | Yes |
| 24 | ENO1 |  |  | 241 | 0.314 | 0.0462 | Yes |
| 25 | ACP1 |  |  | 249 | 0.310 | 0.0477 | Yes |
| 26 | OAZ1 |  |  | 259 | 0.305 | 0.0487 | Yes |
| 27 | RPP21 |  |  | 261 | 0.304 | 0.0518 | Yes |
| 28 | SNHG6 |  |  | 266 | 0.303 | 0.0541 | Yes |
| 29 | ARF1 |  |  | 281 | 0.296 | 0.0536 | Yes |
| 30 | WDR45B |  |  | 288 | 0.295 | 0.0553 | Yes |
| 31 | CDC42 |  |  | 289 | 0.295 | 0.0585 | Yes |
| 32 | POLE3 |  |  | 301 | 0.290 | 0.0588 | Yes |
| 33 | GAPDH |  |  | 320 | 0.286 | 0.0571 | Yes |
| 34 | AIMP1 |  |  | 336 | 0.282 | 0.0562 | Yes |
| 35 | ZC3H15 |  |  | 347 | 0.278 | 0.0566 | Yes |
| 36 | ARF4 |  |  | 348 | 0.277 | 0.0597 | Yes |
| 37 | GUK1 |  |  | 349 | 0.276 | 0.0627 | Yes |
| 38 | RPL15 |  |  | 363 | 0.271 | 0.0622 | Yes |
| 39 | ZFAND1 |  |  | 366 | 0.271 | 0.0647 | Yes |
| 40 | SARS |  |  | 367 | 0.270 | 0.0677 | Yes |
| 41 | COX17 |  |  | 368 | 0.270 | 0.0707 | Yes |
| 42 | SNRPD1 |  |  | 376 | 0.268 | 0.0718 | Yes |
| 43 | PDRG1 |  |  | 378 | 0.268 | 0.0745 | Yes |
| 44 | H2AFZ |  |  | 386 | 0.265 | 0.0756 | Yes |
| 45 | EIF4H |  |  | 389 | 0.264 | 0.0780 | Yes |
| 46 | CCDC90B |  |  | 390 | 0.263 | 0.0809 | Yes |
| 47 | MDH2 |  |  | 393 | 0.262 | 0.0833 | Yes |
| 48 | SEC22B |  |  | 410 | 0.256 | 0.0818 | Yes |
| 49 | TRAPPC1 |  |  | 411 | 0.256 | 0.0846 | Yes |
| 50 | EIF3M |  |  | 419 | 0.253 | 0.0856 | Yes |
| 51 | NDUFB4 |  |  | 427 | 0.252 | 0.0865 | Yes |
| 52 | UQCRH |  |  | 429 | 0.250 | 0.0890 | Yes |
| 53 | HK2 |  |  | 432 | -0.250 | 0.0912 | Yes |
| 54 | CDKL1 |  |  | 434 | -0.250 | 0.0937 | Yes |
| 55 | ALDOA |  |  | 440 | -0.251 | 0.0952 | Yes |
| 56 | DNAJB9 |  |  | 448 | -0.251 | 0.0961 | Yes |
| 57 | PPFIBP1 |  |  | 462 | -0.252 | 0.0954 | Yes |
| 58 | SLC39A7 |  |  | 471 | -0.253 | 0.0960 | Yes |
| 59 | STOX2 |  |  | 487 | -0.254 | 0.0948 | Yes |
| 60 | CTSL |  |  | 492 | -0.254 | 0.0965 | Yes |
| 61 | ZNF791 |  |  | 501 | -0.254 | 0.0972 | Yes |
| 62 | ST3GAL5 |  |  | 519 | -0.255 | 0.0954 | Yes |
| 63 | POU2F2 |  |  | 522 | -0.255 | 0.0977 | Yes |
| 64 | SCAI |  |  | 524 | -0.255 | 0.1003 | Yes |
| 65 | DCUN1D2 |  |  | 558 | -0.257 | 0.0942 | No |
| 66 | DNM1 |  |  | 576 | -0.258 | 0.0925 | No |
| 67 | DCAF12 |  |  | 588 | -0.259 | 0.0924 | No |
| 68 | GPHN |  |  | 625 | -0.261 | 0.0856 | No |
| 69 | NPRL2 |  |  | 646 | -0.262 | 0.0831 | No |
| 70 | FRY |  |  | 661 | -0.263 | 0.0822 | No |
| 71 | TM9SF2 |  |  | 664 | -0.263 | 0.0846 | No |
| 72 | NR6A1 |  |  | 683 | -0.264 | 0.0827 | No |
| 73 | KIF1A |  |  | 719 | -0.266 | 0.0761 | No |
| 74 | EDEM3 |  |  | 730 | -0.266 | 0.0764 | No |
| 75 | UNC50 |  |  | 763 | -0.267 | 0.0707 | No |
| 76 | ANKRD27 |  |  | 768 | -0.268 | 0.0726 | No |
| 77 | ARL6IP5 |  |  | 775 | -0.268 | 0.0740 | No |
| 78 | DNAJC3 |  |  | 780 | -0.268 | 0.0759 | No |
| 79 | FASTKD3 |  |  | 829 | -0.271 | 0.0659 | No |
| 80 | RNF152 |  |  | 856 | -0.272 | 0.0619 | No |
| 81 | FAM219A |  |  | 928 | -0.276 | 0.0457 | No |
| 82 | TNRC6C |  |  | 940 | -0.276 | 0.0458 | No |
| 83 | RRNAD1 |  |  | 971 | -0.278 | 0.0408 | No |
| 84 | GNAZ |  |  | 992 | -0.279 | 0.0385 | No |
| 85 | SSR1 |  |  | 993 | -0.279 | 0.0416 | No |
| 86 | C5orf22 |  |  | 1000 | -0.279 | 0.0431 | No |
| 87 | BTRC |  |  | 1015 | -0.280 | 0.0424 | No |
| 88 | ZNF586 |  |  | 1043 | -0.282 | 0.0382 | No |
| 89 | FKBP11 |  |  | 1062 | -0.283 | 0.0365 | No |
| 90 | AMN1 |  |  | 1066 | -0.283 | 0.0388 | No |
| 91 | GAL3ST4 |  |  | 1157 | -0.288 | 0.0177 | No |
| 92 | UVSSA |  |  | 1164 | -0.288 | 0.0192 | No |
| 93 | HSPA13 |  |  | 1216 | -0.291 | 0.0087 | No |
| 94 | ZNF397 |  |  | 1218 | -0.291 | 0.0116 | No |
| 95 | GOLGB1 |  |  | 1232 | -0.292 | 0.0114 | No |
| 96 | RUFY3 |  |  | 1257 | -0.293 | 0.0081 | No |
| 97 | PRNP |  |  | 1309 | -0.295 | -0.0024 | No |
| 98 | WASF3 |  |  | 1313 | -0.295 | 0.0001 | No |
| 99 | FUT11 |  |  | 1324 | -0.296 | 0.0007 | No |
| 100 | TUBG2 |  |  | 1327 | -0.296 | 0.0034 | No |
| 101 | TADA1 |  |  | 1328 | -0.296 | 0.0067 | No |
| 102 | ELOVL6 |  |  | 1332 | -0.296 | 0.0092 | No |
| 103 | APBB1 |  |  | 1354 | -0.298 | 0.0068 | No |
| 104 | GDPD1 |  |  | 1375 | -0.299 | 0.0048 | No |
| 105 | TSEN2 |  |  | 1398 | -0.300 | 0.0021 | No |
| 106 | CDON |  |  | 1423 | -0.301 | -0.0010 | No |
| 107 | FGF14 |  |  | 1428 | -0.302 | 0.0013 | No |
| 108 | TTC32 |  |  | 1459 | -0.304 | -0.0035 | No |
| 109 | SEC63 |  |  | 1534 | -0.308 | -0.0201 | No |
| 110 | MARS |  |  | 1541 | -0.308 | -0.0183 | No |
| 111 | NIPAL3 |  |  | 1571 | -0.310 | -0.0227 | No |
| 112 | PRR4 |  |  | 1595 | -0.312 | -0.0254 | No |
| 113 | SGK494 |  |  | 1610 | -0.313 | -0.0257 | No |
| 114 | BEX2 |  |  | 1617 | -0.313 | -0.0239 | No |
| 115 | KANTR |  |  | 1626 | -0.314 | -0.0226 | No |
| 116 | CDKN1A |  |  | 1631 | -0.314 | -0.0201 | No |
| 117 | ASAH1 |  |  | 1643 | -0.315 | -0.0196 | No |
| 118 | TM9SF4 |  |  | 1668 | -0.317 | -0.0226 | No |
| 119 | FSD1 |  |  | 1677 | -0.317 | -0.0212 | No |
| 120 | ZNF714 |  |  | 1709 | -0.319 | -0.0261 | No |
| 121 | PGAM5 |  |  | 1711 | -0.319 | -0.0228 | No |
| 122 | SLC25A12 |  |  | 1712 | -0.319 | -0.0192 | No |
| 123 | PDK1 |  |  | 1723 | -0.320 | -0.0184 | No |
| 124 | SCD |  |  | 1737 | -0.321 | -0.0183 | No |
| 125 | PEG3 |  |  | 1754 | -0.322 | -0.0191 | No |
| 126 | LRCH2 |  |  | 1765 | -0.323 | -0.0182 | No |
| 127 | SERINC1 |  |  | 1837 | -0.326 | -0.0338 | No |
| 128 | GLG1 |  |  | 1839 | -0.326 | -0.0304 | No |
| 129 | PDE5A |  |  | 1869 | -0.328 | -0.0346 | No |
| 130 | PXK |  |  | 1871 | -0.328 | -0.0312 | No |
| 131 | CNEP1R1 |  |  | 1883 | -0.329 | -0.0305 | No |
| 132 | DAAM1 |  |  | 1915 | -0.330 | -0.0353 | No |
| 133 | HKR1 |  |  | 1948 | -0.332 | -0.0402 | No |
| 134 | ZNF25 |  |  | 1979 | -0.334 | -0.0446 | No |
| 135 | COG3 |  |  | 1981 | -0.334 | -0.0412 | No |
| 136 | PCDHB2 |  |  | 2011 | -0.336 | -0.0453 | No |
| 137 | RASA4 |  |  | 2013 | -0.336 | -0.0418 | No |
| 138 | DDIT4 |  |  | 2027 | -0.337 | -0.0416 | No |
| 139 | ANKRD46 |  |  | 2028 | -0.337 | -0.0378 | No |
| 140 | MAML3 |  |  | 2034 | -0.337 | -0.0354 | No |
| 141 | HMGCS1 |  |  | 2060 | -0.339 | -0.0384 | No |
| 142 | SEC22A |  |  | 2061 | -0.339 | -0.0347 | No |
| 143 | ACTR1B |  |  | 2078 | -0.340 | -0.0352 | No |
| 144 | DGKE |  |  | 2084 | -0.341 | -0.0328 | No |
| 145 | POLR3E |  |  | 2092 | -0.341 | -0.0309 | No |
| 146 | SLC2A3 |  |  | 2117 | -0.343 | -0.0336 | No |
| 147 | RAB6B |  |  | 2129 | -0.344 | -0.0327 | No |
| 148 | PDPR |  |  | 2176 | -0.347 | -0.0413 | No |
| 149 | SEZ6L2 |  |  | 2251 | -0.353 | -0.0574 | No |
| 150 | SLC38A1 |  |  | 2255 | -0.353 | -0.0543 | No |
| 151 | PGM2L1 |  |  | 2260 | -0.354 | -0.0514 | No |
| 152 | SEC61A2 |  |  | 2286 | -0.356 | -0.0542 | No |
| 153 | BSG |  |  | 2316 | -0.359 | -0.0581 | No |
| 154 | STRIP1 |  |  | 2341 | -0.361 | -0.0606 | No |
| 155 | PFKFB4 |  |  | 2347 | -0.361 | -0.0579 | No |
| 156 | TMEM59L |  |  | 2359 | -0.362 | -0.0569 | No |
| 157 | ENO2 |  |  | 2366 | -0.363 | -0.0544 | No |
| 158 | FBXO38 |  |  | 2370 | -0.363 | -0.0512 | No |
| 159 | KDM5B |  |  | 2402 | -0.365 | -0.0555 | No |
| 160 | TARS2 |  |  | 2449 | -0.369 | -0.0639 | No |
| 161 | TIMM23B |  |  | 2452 | -0.369 | -0.0603 | No |
| 162 | PEX11B |  |  | 2475 | -0.372 | -0.0621 | No |
| 163 | PDIA6 |  |  | 2505 | -0.373 | -0.0658 | No |
| 164 | SLC9A6 |  |  | 2520 | -0.375 | -0.0654 | No |
| 165 | ABAT |  |  | 2521 | -0.375 | -0.0613 | No |
| 166 | KDM5A |  |  | 2563 | -0.379 | -0.0682 | No |
| 167 | GOLPH3L |  |  | 2575 | -0.380 | -0.0669 | No |
| 168 | COPG2 |  |  | 2579 | -0.380 | -0.0635 | No |
| 169 | ZFYVE1 |  |  | 2590 | -0.381 | -0.0620 | No |
| 170 | ATP6AP2 |  |  | 2591 | -0.381 | -0.0577 | No |
| 171 | BBS4 |  |  | 2599 | -0.382 | -0.0554 | No |
| 172 | PAX6 |  |  | 2619 | -0.384 | -0.0562 | No |
| 173 | DACT3 |  |  | 2624 | -0.384 | -0.0530 | No |
| 174 | TMEM175 |  |  | 2645 | -0.385 | -0.0542 | No |
| 175 | ANKRA2 |  |  | 2650 | -0.386 | -0.0509 | No |
| 176 | DHCR7 |  |  | 2654 | -0.386 | -0.0475 | No |
| 177 | ZNF333 |  |  | 2662 | -0.387 | -0.0450 | No |
| 178 | ELOVL4 |  |  | 2667 | -0.387 | -0.0418 | No |
| 179 | HMGCR |  |  | 2673 | -0.388 | -0.0388 | No |
| 180 | DUSP8 |  |  | 2784 | -0.397 | -0.0642 | No |
| 181 | CYP51A1 |  |  | 2826 | -0.402 | -0.0708 | No |
| 182 | COX18 |  |  | 2829 | -0.402 | -0.0669 | No |
| 183 | CBWD3 |  |  | 2833 | -0.402 | -0.0632 | No |
| 184 | ATP1B1 |  |  | 2865 | -0.405 | -0.0671 | No |
| 185 | GOLGA8B |  |  | 2885 | -0.407 | -0.0677 | No |
| 186 | ARMCX4 |  |  | 2896 | -0.408 | -0.0659 | No |
| 187 | TENM1 |  |  | 2915 | -0.410 | -0.0662 | No |
| 188 | ZBTB21 |  |  | 2952 | -0.414 | -0.0714 | No |
| 189 | PRUNE2 |  |  | 2966 | -0.416 | -0.0703 | No |
| 190 | CLCN6 |  |  | 2967 | -0.416 | -0.0656 | No |
| 191 | VWA5A |  |  | 3002 | -0.420 | -0.0702 | No |
| 192 | BTAF1 |  |  | 3036 | -0.425 | -0.0744 | No |
| 193 | ZDHHC6 |  |  | 3047 | -0.425 | -0.0723 | No |
| 194 | GOLGA8A |  |  | 3053 | -0.426 | -0.0690 | No |
| 195 | SLC48A1 |  |  | 3094 | -0.430 | -0.0750 | No |
| 196 | FICD |  |  | 3111 | -0.432 | -0.0745 | No |
| 197 | ANKS1A |  |  | 3134 | -0.435 | -0.0756 | No |
| 198 | HINFP |  |  | 3141 | -0.436 | -0.0724 | No |
| 199 | PTPRN |  |  | 3149 | -0.437 | -0.0694 | No |
| 200 | ZNF234 |  |  | 3182 | -0.441 | -0.0732 | No |
| 201 | SV2A |  |  | 3186 | -0.441 | -0.0691 | No |
| 202 | HSPA5 |  |  | 3193 | -0.442 | -0.0658 | No |
| 203 | EXD2 |  |  | 3211 | -0.444 | -0.0654 | No |
| 204 | MSMO1 |  |  | 3241 | -0.450 | -0.0683 | No |
| 205 | PTPRG |  |  | 3246 | -0.451 | -0.0643 | No |
| 206 | GGA2 |  |  | 3249 | -0.451 | -0.0599 | No |
| 207 | NR2C1 |  |  | 3268 | -0.454 | -0.0597 | No |
| 208 | KLHL28 |  |  | 3280 | -0.456 | -0.0576 | No |
| 209 | ANKZF1 |  |  | 3302 | -0.460 | -0.0582 | No |
| 210 | GAP43 |  |  | 3308 | -0.462 | -0.0544 | No |
| 211 | TVP23B |  |  | 3323 | -0.466 | -0.0530 | No |
| 212 | ABCA5 |  |  | 3377 | -0.475 | -0.0620 | No |
| 213 | NAPB |  |  | 3389 | -0.479 | -0.0597 | No |
| 214 | OLFM1 |  |  | 3398 | -0.480 | -0.0565 | No |
| 215 | ZDBF2 |  |  | 3415 | -0.483 | -0.0554 | No |
| 216 | PCLO |  |  | 3417 | -0.483 | -0.0503 | No |
| 217 | DGCR2 |  |  | 3431 | -0.487 | -0.0484 | No |
| 218 | MTHFR |  |  | 3509 | -0.511 | -0.0636 | No |
| 219 | TSPAN7 |  |  | 3528 | -0.520 | -0.0627 | No |
| 220 | HOMER2 |  |  | 3531 | -0.521 | -0.0574 | No |
| 221 | ZFHX4 |  |  | 3540 | -0.524 | -0.0538 | No |
| 222 | KIAA1958 |  |  | 3557 | -0.530 | -0.0522 | No |
| 223 | TARBP1 |  |  | 3620 | -0.560 | -0.0627 | No |
| 224 | CXADR |  |  | 3670 | -0.591 | -0.0694 | No |
| 225 | PNMA2 |  |  | 3720 | -0.633 | -0.0756 | No |
| 226 | SLC35F1 |  |  | 3724 | -0.635 | -0.0694 | No |
| 227 | MEIS1 |  |  | 3727 | -0.637 | -0.0628 | No |
| 228 | STC1 |  |  | 3770 | -0.702 | -0.0664 | No |
| 229 | SCG2 |  |  | 3786 | -0.718 | -0.0625 | No |
| 230 | MIAT |  |  | 3833 | -0.794 | -0.0661 | No |
| 231 | KCNQ1OT1 |  |  | 3866 | -0.885 | -0.0649 | No |
| 232 | SDK2 |  |  | 3868 | -0.898 | -0.0551 | No |
| 233 | NEAT1 |  |  | 3880 | -1.005 | -0.0469 | No |
| 234 | IGFBP5 |  |  | 3895 | -1.082 | -0.0387 | No |
| 235 | NRP2 |  |  | 3905 | -1.241 | -0.0273 | No |
| 236 | IGFBP2 |  |  | 3917 | -1.404 | -0.0146 | No |
| 237 | MALAT1 |  |  | 3924 | -1.583 | 0.0014 | No |
Table: GSEA details [plain text format]

  

Fig 2: RICHARDS\_NOWAKOWSKI\_SCIENCE\_2017\_GLYC\_UPREG: Random ES distribution      
 Gene set null distribution of ES for **RICHARDS\_NOWAKOWSKI\_SCIENCE\_2017\_GLYC\_UPREG**
